# How we do: optimizing bone marrow biopsy logistics for sign-out within 2 days

**DOI:** 10.1007/s12308-016-0270-y

**Published:** 2016-03-05

**Authors:** I. de Laak–de Vries, A. G. Siebers, L. Burgers, C. Diepenbroek, M. Link, P. Groenen, J. H. J. M. van Krieken, K. M. Hebeda

**Affiliations:** Department of Pathology 824, Radboud University Medical Center, P.O. Box 9101, 6500 HB Nijmegen, The Netherlands

**Keywords:** Bone marrow examination, Decalcification technique

## Abstract

Since the introduction of fast diagnostic tracks in many areas of oncology, the traditional processing of bone marrow biopsies (BMB), requiring either resin embedding or lengthy fixation and decalcification, is due to an upgrade. Thanks to a growing number of new commercially available tissue processors, microwave-enhanced processing is becoming a standard tool in the pathology laboratory, allowing rapid fixation and decalcification of BMB with preserved morphology and antigens. In this short report, we describe the use of a commercially available EDTA-based decalcification fluid (USEDECALC, Medite, Orlando, USA) in combination with the LOGOS J (Milestone, Bergamo, Italy), a closed microwave-enhanced tissue processor, for overnight fixation, decalcification, and paraffin impregnation of the BMB. This allows next-day reporting without impaired morphology or immunohistochemistry, and even improved DNA quality of the BMB.

## Introduction

Many systemic diseases, including auto-immune and infectious diseases, but also various malignancies present or originate in the bone marrow. Despite developments in immune flow cytometry and molecular diagnostics of blood and bone marrow, the trephine bone marrow biopsy (BMB) remains a crucial diagnostic, staging, and prognostic tool in hematopathology. Immunohistochemistry and molecular techniques on the BMB complete traditional morphology [1, 2], requiring preservation of antigens, DNA, and RNA. Due to the growing importance of single day stays in the outpatient clinics, the pressure to shorten turnaround times of BMB increases. This requires redesigning the process of fixation and decalcification of BMB, which traditionally required up to 5 days.

The combination of the hard, bony structures, and the enclosed delicate cells with many different nuclear and cytoplasmic structures, containing enzymes that have to be preserved, make processing of BMB a challenge. Over the years, various fixatives have been developed to improve morphology or speed up the fixation from the traditional 24–48 h required with standard formalin, but these fixatives are not necessarily suitable for immunohistochemistry and molecular techniques. Therefore, formalin-based fixatives are still preferred in most pathology laboratories. Although resin embedding allows cutting of the bone with optimal preservation of cytological details, decalcification followed by paraffin embedding is nowadays mostly preferred, since the loss of cellular detail by decalcification can be compensated by cytomorphology of the aspirate, supplemented with immunohistochemistry on the BMB.

Both decalcification and resin embedding slow the processing of BMB considerably, requiring up to two additional days. Acidic fluids such as formic acid were introduced to speed up the decalcification process, however, compromising morphology, immunohistochemistry, enzyme reaction, and DNA/RNA quality. As an alternative, 24- to 48-h formalin fixation, followed by ultrasound [3] or microwave [4] accelerated decalcification in EDTA, was introduced, leading to an improved turnaround time and good diagnostic quality. We further improved our previous BMB protocol to reach a turnaround time of 2 days (Table 1) with equal morphological, immunohistochemical, and DNA quality.

**Table 1:** Summary of the protocol for rapid microwave-enhanced processing of the bone marrow biopsy

Day 1	9am–4pm	Arrival of BMB at department of pathology	In Burckhardt’s fixative
	5pm	Start LOGOS J:	Automated, in closed container
		Fixation in formaldehyde	
		Decalcification in USEDECALC	
		Dehydration	
		Paraffin impregnation	
Day 2	7am	Embedding BMB in paraffin block	
		Section the block	4-μm sections
	11am	Automated H&E stain, delivery to pathologist	
	1pm	Automated conventional histochemical staining	Giemsa, PAS, Perls, Laguesse, Leder
	4pm	Automated immunohistochemistry	CD34, CD117, CD61, CD20, CD3, and CD138
	5pm	Sign-out of the cases, report available in electronic patient file	

### The biopsy

**Fig. 1:**
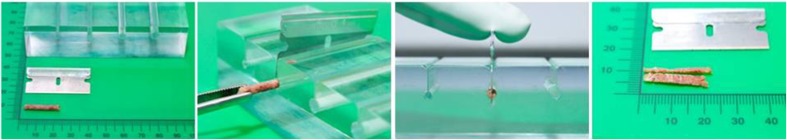
Splicing of the bone marrow biopsy in a Perspex mold

The thickness and bone density of the tissue determine the minimum time for fixation and decalcification. Our new, overnight protocol allows no more than 2-mm-thick bone samples. Therefore, we use a Perspex mold (Klinipath, Duiven, Netherlands, Fig. 1) to splice the BMB lengthwise. This requires 3–4-mm-thick, good-quality biopsies. In our hospital, hematologists and physician assistants use Macom 8G needles (Lettix, Apeldoorn, Netherlands). The splicing of the BMB enabled us to directly compare the results of our previous and new protocol by processing each half with a different protocol. No cutting artifacts were observed after splicing procedure. For clinical validation, 198 BMB were thus compared for routine histochemical stains and a set of relevant immunohistochemical stains. All illustrations in this report are comparisons from this set of patients.

### Fixation

Traditionally, we have used Burkhardt’s fixative, a 12 % buffered formaldehyde solution supplemented with methanol and glucose, for many years because of the better morphology than achieved with buffered formaldehyde alone, when using our previous protocols. We still receive the BMB in Burkhardt’s, but the current protocol is performed after exchanging this fixative for buffered formaldehyde.

### Decalcification and processing

**Fig. 2:**
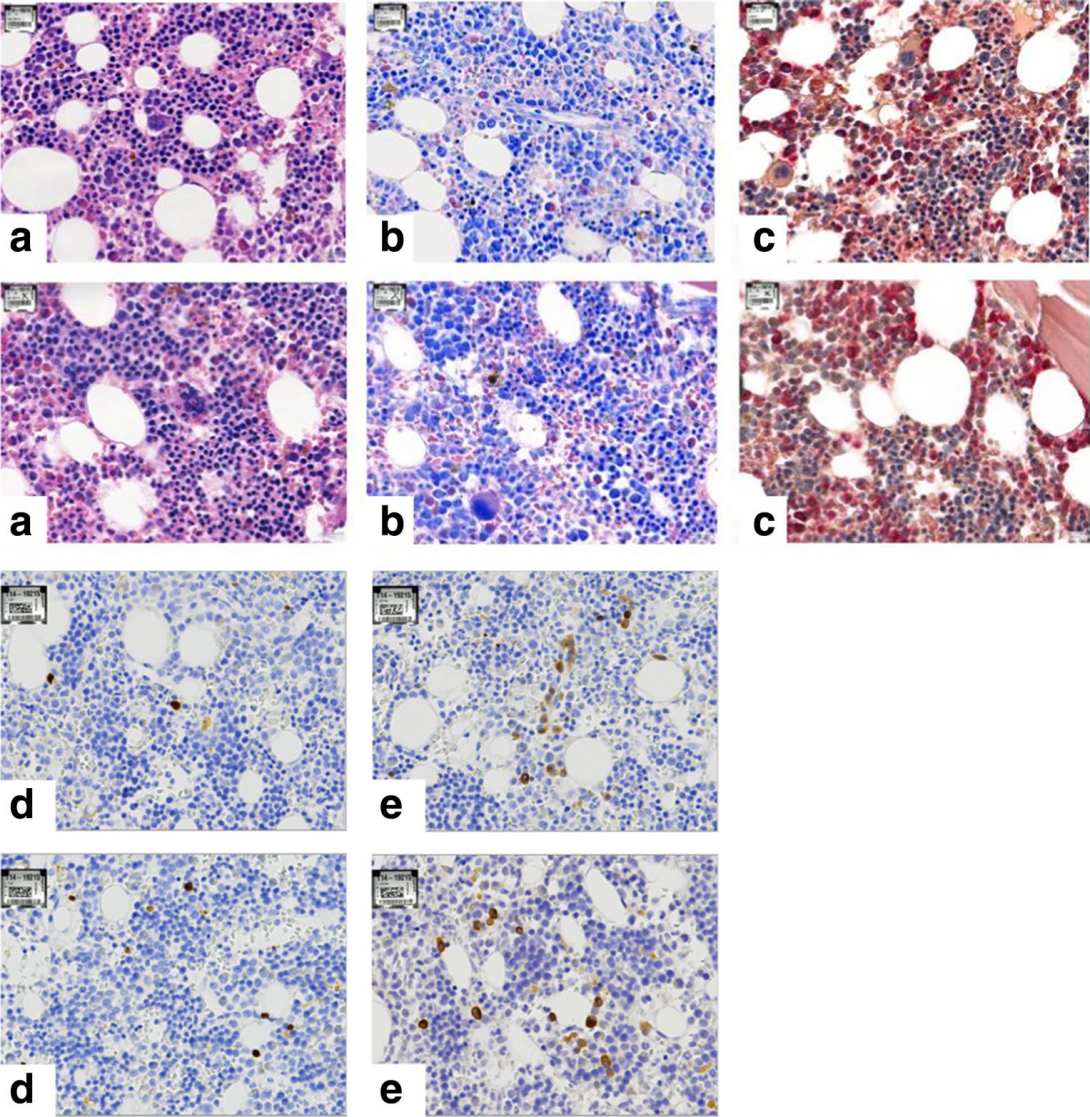
Routine histochemical staining results for H&E (**a**), Giemsa (**b**), Leder (**c**), TdT (**d**), and CD79a (**e**) stain with our previous protocol and the rapid processing protocol

The BMB are then processed in the LOGOS J (Milestone, Bergamo, Italy), a fully automated system that performs microwave-enhanced fixation, decalcification, and paraffin impregnation in a closed system. This enables overnight protocols (Table 1). For rapid decalcification, we use a chelating, EDTA-based solution (USEDECALC, Medite, Orlando, USA). Compared to other commercially available decalcifying solutions that we have tested, this gave the best results for morphology and DNA yield. Another important factor that determines the results, notably of the Leder (chloracetate esterase) stain since this requires an intact enzyme, is the maximum temperature that is used during the whole processing. Restricting the temperature to 60 °C allows enzyme stains (Fig. 2c; Leder staining) with a slightly better contrast than with our previous protocol. The technical details of the program are presented in Table 2.

**Table 2:** Technical details of the program steps of the rapid processing protocol using LOGOS J

Step		Duration
1	Neutral buffered formalin (4 % formaldehyde)	15 min, 37 °C
		2 h 15 min, 37 °C
2	USEDECALC	10 min, 40 °C
		6 h 50 min, 40 °C
3	Water rinsing	30 min RT
4	Flush mix (ethanol 60 %)	2 min RT
5	Ethanol 100 %	2 min, 50 °C
6	Ethanol 100 %	3 min, 50 °C
7	Ethanol 100 %	10 min, 50 °C
		30 min, 55 °C
8	Isopropanol	10 min, 55 °C
		30 min, 55 °C
9	Wax/paraffin	10 min, 60 °C
		90 min, 60 °C

### Sectioning and staining

The LOGOS J is ran from 5 pm to 6.30 am the next morning. Thereafter, the BMB are embedded in paraffin blocks, and 4-μm sections are routinely stained with Hematoxylin-Eosin (H&E), Giemsa, Laguesse, PAS, Perls, and in selected cases Leder solutions. Of these stains, only the protocol for the Giemsa staining had to be slightly adapted since the colors were not as intense as in our previous protocol. Therefore, we have to vary the differentiation time somewhat from case to case (Fig. 2b; comparison of Giemsa stain). Iron and fibers are equally preserved.

### Immunohistochemistry and in situ hybridization

Many BMB are performed for clinical suspicion of a myelodysplastic syndrome. Our standard immunohistochemical protocol for this evaluation consists of antibodies against CD34, CD117, CD61, CD20, CD3, and CD138. The incubation protocols did not need any changes after introduction of the rapid decalcification, and immunohistochemistry performs equally well (Fig. 2d, e). We use the Lab Vision Autostainer 480 (ImmunoLogic, Duiven, The Netherlands) for immunohistochemistry with a 2-h protocol, allowing sign-out of the cases the same day as the routine stains become available.

Also, the protocols for in situ hybridization (ISH) for EBER and fluorescence ISH with probes for BCL2, BCL6, CCDN1, and MYC perform equally well after the rapid decalcification (data not shown).

### DNA extraction

**Fig. 3:**
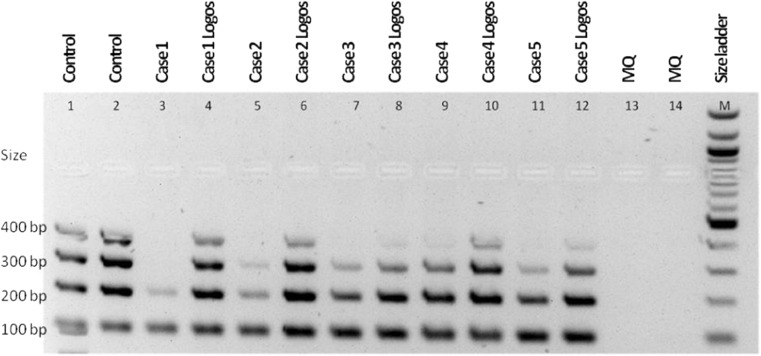
Comparison of DNA quality depicting DNA fragment size of spliced bone marrow biopsies processed with the standard and the rapid decalcification (Logos) protocol, showing improved DNA quality after rapid decalcification

For immunoglobulin gene and T cell receptor gene rearrangement studies, the DNA is extracted from 20-μm slides using proteinase K treatment and ethanol precipitation of the genomic DNA. Subsequent purification of the DNA is performed with a QIAamp DNA Micro Kit (QIAGEN, Hilden, Germany). To compare the integrity of the retrieved DNA with the previous and new protocols, the concentration was adjusted to 50 ng/μl and a size ladder PCR was performed [5]. The DNA was run on an agarose gel by electrophoresis. The maximum size of the bands of both protocols was compared with the size ladder PCR products (Fig. 3). DNA degradation is less with the new rapid processing, allowing detection of larger products with this method in six of ten tested biopsies.

## Discussion

We optimized different steps of our decalcifying protocol during the past years to arrive at our current protocol. Indeed, alternative agents for rapid decalcification at room temperature with acceptable DNA and RNA results have been reported. These are mainly combinations with 14 % EDTA or formic acid (for example, Formical-4 (formic acid/EDTA/formaldehyde, Decal Chemical Corporation, Tallman, NY), Formical-2000 (EDTA/formic acid, Decal Chemical Corporation, Tallman, NY), Immunocal (formic acid, Decal Chemical Corporation, Tallman, NY), and Surgipath Decalcifier I (formic acid/methanol/formaldehyde, Leica Biosystems) [6]). On the other hand, hydrochloric and nitric acid-based agents are not recommended or show clearly worse results in several studies (for example, Nitrical (nitric acid, Decal Chemical Corporation, Tallman, NY), IMEB (HCL/formic acid, IMEB, San Marcos, Calif), Surgipath Decalcifier II (HCL/EDTA, Leica Biosystems), Overnight bone decal (EDTA/HCL, Decal Chemical Corporation, Tallman, NY), Decal Stat (HCL/EDTA, Decal Chemical Corporation, Tallman, NY), and RDO Gold (Hydrochloric acid based, Apex Engineering Products Corporation, Aurora, IL) [6, 7]).

In conclusion, by adjusting our protocol for the processing of BMB, we successfully reduced the processing time of BMB, allowing sign-out within 24 h of receipt of the biopsy without compromising the quality of morphology and antigen detection and improved DNA quality.
